# Are Usual and Customary Charges Reasonable?

**DOI:** 10.5811/westjem.2016.10.32683

**Published:** 2016-10-25

**Authors:** R. Myles Riner

## Abstract

An editorial about the use of usual and customary charges for out-of-network benefit determinations.

In State legislative offices throughout the country where the issue of out-of-network (OON) physician charges, balance billing, and plan benefits are being debated, the constant refrain from health plan representatives is that usual and customary (U&C) charges are an “unacceptable standard” for OON benefits. Nope, won’t consider it, won’t even discuss it: U&C charges are “off the table.” Aside from the fact that, when one side in a negotiation takes something off the table at the start, it really is no longer a negotiation: is it reasonable to eliminate U&C charges from consideration?

It wasn’t that long ago that health plans would allow (pay) a benefit based on the lesser of the physician’s full charge or the 70^th^ or 80^th^ percentile of U&C charges based on the Ingenix database. Things changed when the Attorney General (AG) of New York got wind of the fact that health plans were deliberately manipulating the claims data^1^ that generated this United Healthcare-owned database in order to cheat enrollees out of hundreds of millions of dollars in benefits for OON services, and sued several plans for this abusive tactic. Suddenly, having been caught with their fingers in the cookie jar, commercial health plans almost universally and simultaneously decided to abandon the U&C charge standard for OON benefits. The AG required several of these plans to fund the development of a new, independent U&C charge database called FAIR Health;^2^ but since these plans were limited in their ability to manipulate the new database, most decided to rely on other standards where state regulations allowed. Most of the new standards for OON benefits are either based on a percentage of Medicare rates or on the plan’s own highly arbitrary, black-box, “usual, customary and reasonable” rates,^1^ all of which are considerably lower than (often less than half of) the 70^th^ percentile of U&C charges. The plans rationalize this new approach in the following ways:

- It is necessary to keep premiums down.
- U&C charges are too high because there is nothing that keeps physicians from overcharging for their services, or consistently raising fees.
- Outlier physician charges distort U&C charge databases.
- It is a way to encourage enrollees to preferentially use in-network physicians.

Let’s look at these arguments. Of course, limiting plan benefit payouts might keep premiums down, but so would limiting plan profits; yet profits and premiums have risen in lockstep.^3^ Also, there is no evidence that limiting OON benefits has kept premiums from increasing, and in many cases enrollees are not getting the benefits that their premiums are supposed to secure. The argument that there are no economic factors keeping physician charges in check ignores the very real competitive forces that constrain physician charges.^4^ Hospitals that contract with physicians for services want their physicians to be sensitive to their market. Physicians who charge high prices and refuse to contract with plans and discount their charges to health plan enrollees will have difficulty filling their offices or surgery schedules unless their skills, reputations, and services are exceptional and in great demand. It is true that outlier charges can distort U&C charge databases when the survey areas are small, or when large, high-charging physician groups dominate in their market; but these impacts can be easily mitigated by expanding the size of survey areas and maximizing the number of claims included. Lastly, as plans shrink the size of their networks to include fewer providers, enrollees may be forced outside of these narrow networks to obtain needed services from the most qualified physicians,^6^ and they shouldn’t be excessively penalized for doing so. Many narrow networks deliberately avoid contracting with emergency care providers,^1^ relying instead on emergency departments and Emergency Medical Treatment and Labor Act (EMTALA) regulations to ensure their enrollees have access to emergency care, forcing these physicians to attempt to get reasonable payment after the fact as OON providers.

The concept behind using a U&C charge database for OON benefits is that these charges reflect the various forces that define the reasonable market value of these services, including the cost of providing them. A physician who is providing services outside of a health plan network is usually not receiving any of the other considerations from a health plan in return for discounting their services to the plan’s enrollees. These considerations might include a large referral base, faster payment, fewer denials of coverage, direct to provider payments, etc. Taking a large sampling of claims from physicians and looking at the range of charges (fees) for these services, then lopping off the highest 20 or 30% of these as “too far above the mean,” allows for the identification of a “reasonable range of fees” that reflect the market value of these services. This is why this approach was used by plans in the past to determine what the reasonable benefit should be for OON services. Some plans still do this, but now most plans have decided they need to redefine “reasonable market value” to mean “whatever we think is reasonable.”

You could argue that the market for physician services isn’t really an open, fair, and competitive market, and you might be right in many areas of the country, but this is why the top 20% or 30% of charges are excluded from the “reasonable” standard for OON benefits. There is nothing logical or reasonable about allowing plans to make this determination independently, especially if physicians are prohibited by law or regulation from seeking to recover more than the amount that the plan “allows” for OON services. If plans want to set fees, they should be forced to go through the equivalent of a public utilities commission process;^8^ otherwise they are using the government to steal those services from providers at an unwarranted discount. If anything should be off the table in these negotiations for an OON benefit standard and balance billing legislation, it should be offering plans a license to steal.
